# Climate Donations Inspired by Evidence-Based Fundraising

**DOI:** 10.3389/fpsyg.2022.768823

**Published:** 2022-03-07

**Authors:** Ren Ryba, Matthew J. Dry, Sean D. Connell

**Affiliations:** ^1^Southern Seas Ecology Laboratories, School of Biological Sciences, The University of Adelaide, Adelaide, SA, Australia; ^2^School of Psychology, The University of Adelaide, Adelaide, SA, Australia

**Keywords:** communication, conservation, effective altruism, non-profit, philanthropy

## Abstract

Everyone has an opportunity to contribute to climate solutions. To help people engage with this opportunity, it is critical to understand how climate organizations and fundraisers can best communicate with people and win their financial support. In particular, fundraisers often rely on practical skills and anecdotal beliefs at the expense of scientific knowledge. Fundraisers could be motivated to achieve a substantial boost in funding for climate solutions, if there is evidence of the financial gains that science-based fundraising makes available. In this Perspective, we provide a preliminary foray into such evidence. We bring together findings from philanthropic research and climate psychology to identify what factors can help captivate donors. Then, through an experimental study of a charitable appeal for a climate charity, we show how putting these factors into practice may contribute toward an increase in donated money. This provides optimism that evidence-based fundraising can inspire donors to contribute much-needed resources toward climate solutions.

## Introduction

When it comes to climate solutions, non-profit organizations have a vital role to play (Osuri, 2010). These charities further climate research and policy by funding research into new technologies, developing mitigation strategies, and educating the public (Osuri, 2010; Nisbet, 2018), particularly important roles given the failure of developed nations to deliver public money (Roberts et al., 2021). To perform these roles in society, charities generally rely on donations from the public (Yen et al., 1997; Verssimo et al., 2018). However, there remains a chasm between the current size of the climate non-profit sector and the resources that are needed to effectively confront climate change (Yeo, 2019). So, for climate charities to maximize their impact, it is critical to understand what motivates people to engage with them (Ryba and Connell, 2020).

As fundraising remains an emerging profession, many fundraisers and the charities they support adopt the mind-set that fundraising is more of an art than a science (Cremades and Corcoran, 2016; Phillips, 2016). Fundraisers often emphasize practical skills and anecdotal beliefs at the expense of theoretical and empirical knowledge (Lindahl and Conley, 2002; Bekkers and Wiepking, 2010; Aldrich, 2016). This is despite the inroads that have been made in the scientific literature into how non-profit organizations can use messages to increase their appeal to donors (Bekkers and Wiepking, 2010; Whillans, 2016) and, separately, what drives people’s concern for climate change (Weber, 2010, 2016; Center for Research on Environmental Decisions [CRED] and EcoAmerica, 2014). For fundraising to meaningfully contribute to urgently needed climate solutions, organizations have the opportunity to adopt an evidence-based perspective on how to engage with donors. And for the organizations that do so, the financial rewards may be substantial (Oppenheimer and Olivola, 2011).

In particular, at the center of successful fundraising campaigns is an inspiring message to donors (Bekkers and Wiepking, 2010; Whillans, 2016). Crafting an inspiring message is cost-effective for climate charities, which makes this an accessible, tractable way to maximize the impact of fundraising efforts in this resource-scarce sector (Ramutsindela et al., 2013; Waldron et al., 2013)—all it requires is the motivation to put empirical findings into practice (Whillans, 2016).

The non-profit sector can potentially unlock millions of dollars for climate solutions, if there is evidence of the financial gains that science-based fundraising makes available. Here, we show how such evidence may be provided. We bring together findings from philanthropic research and climate psychology to identify what factors can help captivate donors. Then, through an experimental study of a charitable appeal for a climate charity, we show how putting these factors into practice may cause an increase in donated money.

## The Science of Climate Charity

At the interface of environment and fundraising is an emerging literature on how to spur financial support for environmental non-profits (Yen et al., 1997; Bulte et al., 2005; Israel, 2007; Markowitz et al., 2013; Vollan et al., 2017; Lundberg et al., 2019, 2020; Nelson et al., 2019). Field, laboratory and online studies have identified a range of factors that encourage charitable donations to environmental causes in scenarios involving real money (Christie, 2007; Uehleke and Sturm, 2017; Shreedhar and Mourato, 2019). For example, a donor might give more money if a message evokes emotions rather than social norms (Bergquist et al., 2020); if a message highlights humanity’s responsibility (Shreedhar and Mourato, 2019); if a message emphasizes charismatic or flagship species (Thomas-Walters and Raihani, 2017; Verssimo et al., 2018; Shreedhar and Mourato, 2019); if a message emphasizes threatened species (Veríssimo et al., 2017); if a message focuses on the non-human beneficiaries of donations (Batavia et al., 2018); or even if a message features amusing memes (Lenda et al., 2020).

More specifically, some researchers have begun to investigate the components of an effective message in the context of real-money donations for climate solutions (Löschel et al., 2013). Here, a donor may make give more money if a message emphasizes the impact of anthropogenic climate change, rather than extreme weather events (Ellis et al., 2016); if a message features scientific information from experts about the impacts of climate change (Milinski et al., 2006); if the location of the mitigation is made salient (Diederich and Goeschl, 2018); if the message emphasizes the impact on incomes of future generations (Svenningsen, 2019); if the message is framed as doing good, rather than undoing harm (Blasch, 2014); and possibly if a message highlights social norms (Löschel et al., 2017; Goeschl et al., 2018).

However, these studies are relatively few, and they represent only a subset of the many components of an effective message that have been identified by research on fundraising and, separately, public engagement with climate (Bekkers and Wiepking, 2010; Center for Research on Environmental Decisions [CRED] and EcoAmerica, 2014; Weber, 2016, 2010; Whillans, 2016). So, if climate charities are to make the most of their resources, there is a potentially lucrative opportunity to take advantage of these complementary bodies of literature. Here, we show the potential monetary gains that may arise from doing so, through an experimental study involving real donors giving real money to a climate charity.

## Crafting Captivating Messages: An Experimental Study

A climate organization that engages with the literature on effective messaging may receive significant financial returns. To provide preliminary evidence on these financial benefits, we now turn to our experimental study.

### Data Collection

We created three textual messages that each aimed to solicit donations for a climate organization, Coalition for Rainforest Nations^1^ (Halstead, 2018). The messages were the same in content and logical flow, but they differed systematically in eight components known to influence the effectiveness of charity messages or climate messages (Ryba et al., 2021). This way, we could assess how the different combinations of message characteristics influence the money donated. The combinations of each of the eight components present in each of the three messages are described in Table 1. The eight components themselves were:

**TABLE 1:** The eight components used to craft messages of different impact levels.

Message characteristic	Meaning	Level in high-impact message	Level in med-impact message	Level in low-impact message
Impact	Does the message state the concrete impact of donating?	A concrete measure of the averted carbon dioxide emissions per dollar donated.	A statement that emissions are averted, but with no concrete measure.	A statement that emissions are averted, but with no concrete measure.
Motives	Does the message invoke altruistic or self-interested motives?	A statement that a donation will help preserve the environment.	A statement that a donation will help preserve the environment.	A statement that a donation will give a feeling of satisfaction.
Endorsement	Is the charity endorsed by an authority figure?	An endorsement by a policy researcher from a well-known university.	An endorsement by a policy researcher from a well-known university.	No endorsement by an authority figure.
Co-benefits	Does the message mention positive side effects of donating?	A statement that donations also increase employment in developing countries.	No mention of positive side effects of donating.	No mention of positive side effects of donating.
Frame	Is the message framed in terms of climate or a different issue?	Framed in terms of climate change.	Framed in terms of climate change.	Framed in terms of air pollution and its impact on human health.
Proximity	Does the message focus on consequences of the issue that are nearby in space and time?	Emphasis of consequences in the same country and year of the study.	Emphasis of consequences in a different continent and future century.	Emphasis of consequences in a different continent and future century.
Social norms	Does the message mention how the reader’s peers feel about the issue?	A statement that university students are concerned about the issue.	No mention of university students.	No mention of university students.
Growing risk	Does the message emphasize that the issue is increasing in severity?	A statement that the risk is growing more urgent each year.	No mention of the growing risk.	No mention of the growing risk.

• *Impact*. A charity message may be more engaging if that message emphasizes the concrete effects that a donation would bring about – in other words, the difference that the donor can make (Bekkers and Wiepking, 2010; Whillans, 2016).
• *Motives*. Research has suggested that readers may donate more if a charity message highlights benefits to donors, such as tax benefits or the opportunity to leave a legacy. However, there is a danger that self-interested motives can conflict with altruistic motives (Bekkers and Wiepking, 2010; Zaval et al., 2015; Whillans, 2016).
• *Endorsement*. Some research has found that readers may engage more with charity messages if those messages highlight the endorsement of an authority or public figure (Bekkers and Wiepking, 2010; de Vries and Lubart, 2019).
• *Co-benefits*. A number of studies have found that climate messages may be more effective if they emphasize the fact that acting on climate change may bring co-benefits. For example, a message may emphasize economic benefits that also arrive by action on climate (Maibach et al., 2008; Markowitz and Shariff, 2012; van der Linden et al., 2015; Weber, 2016; Roser-Renouf and Maibach, 2018; Ballew et al., 2019).
• *Frame*. Messages may garner greater support if the content does not discuss climate directly, but instead uses a non-climate frame such as air pollution or health. The effectiveness of this choice also depends on the relevance of the issue (Whitmarsh et al., 2013; Walker et al., 2018).
• *Proximity*. Many research studies have suggested that messages are more engaging when they emphasize consequences that are nearby in space and time. This has been studied in both charitable donations and concern for climate change. However, results are often complex and context-specific (Spence et al., 2012; Evans et al., 2014; Milfont et al., 2014; Stoknes, 2014; Brügger et al., 2015a,b; McDonald et al., 2015; van der Linden et al., 2015; Wiest et al., 2015; Rickard et al., 2016; Weber, 2016; Everuss et al., 2017; Jones et al., 2017; Singh et al., 2017; Brugger and Pidgeon, 2018; Johannsen et al., 2018; Lee et al., 2018; Roser-Renouf and Maibach, 2018; Schuldt et al., 2018; Chen, 2019; Chu and Yang, 2019; Kim and Ahn, 2019; Mildenberger et al., 2019; Romero-Canyas et al., 2019; Wang et al., 2019).
• *Social norms*. Some studies have found evidence that readers express greater concern for climate change if a message mentions that the readers’ peers are also concerned (Gifford, 2011; Kahan et al., 2012; Markowitz and Shariff, 2012; Stoknes, 2014; van der Linden, 2015; van der Linden et al., 2015; Ballew et al., 2019).
• *Growing risk*. Messages that emphasize that the issue of climate change is increasing in severity may increase reader concern (Krosnick et al., 2006; Maibach et al., 2008, 2010; Myers et al., 2012; Hornsey et al., 2016; Berger et al., 2019; Chen, 2019).

The three messages, which were constructed using systematic combinations of the above characteristics, were designated as high, medium, and low impact based on findings from existing research. The combinations of characteristics present in each of the three messages are summarized in Table 1. These messages constitute the experimental treatment in this study (see Supplementary Information for full messages).

The core outcome variable in this study is the amount of money donated to the designated charitable organization. We recruited participants from undergraduate science classrooms. We asked each participant to read one of the three messages, assigned at random via an online survey platform (SurveyMonkey). Each participant was informed, via the survey page, that we were giving them $10 in cash. They were offered the opportunity to donate some amount of this cash to the charity. They were told that they could donate any amount from $0 to 10, and that they would keep any money they did not donate.

To correct for differences in donations due to personal factors (Bekkers and Wiepking, 2011), we also collected data on their characteristics, including their demographics, beliefs, and worldviews, using a survey. At the end, participants received the money they chose to keep, and the money they chose to give was donated to the charity as a lump sum. Participants were also offered the chance to give general feedback. All donations and survey responses were anonymous. Participants were not made aware of the experimental manipulation until after the experiment.

We approached three classrooms, consisting of 79 students, for participation. We selected this number as 55 participants, allowing for 70% completion, achieved a power above 0.95 given an R-squared value of 0.20 and a significance level of 0.05 for multiple regression with 5 predictors (Cohen, 1992). All 79 students chose to participate, and 70 participants (24 high-impact, 24 medium-impact, 22 low-impact) gave complete responses. We only analyzed complete responses, as required by principal component analysis.

Data was collected during three sessions in March and April 2020. The first two sessions were conducted in university laboratories in Adelaide, Australia during teaching hours. The third session (15 participants) was conducted online, since university campuses closed due to the COVID-19 pandemic in between sessions of data collection. Participants in the online session were given a version of the experiment where the money was hypothetical only. Existing research shows that hypothetical rewards and real rewards can yield similar findings (Kühberger et al., 2002; Locey et al., 2011), although divergences have been documented (Vlaev, 2012). We include the hypothetical participants in the statistical model, but also generate a model where they are excluded to examine the effects of this decision (see Discussion).

The experiment and survey were approved by the School of Psychology Human Research Ethics Committee at the University of Adelaide (approval number: H-2020/06).

### Statistical Analysis

We generated linear regression models to assess how donation size was affected by the message and the variables corresponding to the demographics, worldviews, and political beliefs. However, many of those latter variables were highly correlated. To transform these correlated donor characteristics into a set of uncorrelated variables, we applied principal component analysis (PCA). This produced a set of principal components (PCs) that each consisted of a linear combination of donor characteristics. We retained the first three components (PC1, PC2, PC3) based on the criterion of Lott (1973), applicable for principal component regression as performed here (Jolliffe, 2002). On the first component (PC1), a lower position corresponded to the political left, support for progressive parties, concern about climate change, and an egalitarian worldview; a higher position corresponded the political right, support for conservative parties, less concern about climate change, and a hierarchical worldview. On the second component (PC2), a lower position corresponded to younger age, less financial security, and an individualist worldview; a higher position corresponded to older age, greater financial security, and a communitarian worldview. On the third component (PC3), a lower position corresponded to lower religious beliefs, older age, and an individualist worldview; a higher position corresponded to greater religious beliefs, younger age, and a communitarian worldview. We note that these are only some of the items that compose each of the PCs; for the visualization, see Supplementary Information.

We generated linear regression models to assess how donation size was affected by the message and the covariates, as represented by the retained PCs (Table 2). We allowed for interaction terms between the message and each PC, as interactions between message characteristics and donor characteristics have been found in previous research (McDonald et al., 2015; Rickard et al., 2016; e.g., Kim and Ahn, 2019).

**TABLE 2:** Linear regression model for the effects of message impact and personal characteristics on money donated.

	Donation			
Predictors	Estimates	std. Error	*t*	*p*
Intercept	14.83	5.03	2.95	**0.005** *
Impact	−5.46	2.37	−2.30	**0.025** *
PC1	−3.67	4.42	−0.83	0.409
PC2	−9.28	6.10	−1.52	0.133
PC3	−8.68	6.14	−1.41	0.162
PC1*Message	2.74	2.46	1.12	0.268
PC2*Message	6.75	3.14	2.15	**0.035** *
PC3*Message	4.48	2.75	1.63	0.108
Observations df	70 62			
R^2^/R^2^ adjusted	0.157/0.062			
AIC	381.426			

*p < 0.05.

In our models, we expressed message-impact as a continuous variable. We encountered no need to restrict the response variable to between $0 and 10, as the linear model did not make predictions outside this range. Finally, to express results in a way that is meaningful to a charity organization, we used the models to predict the donation at each message impact, given mean values of PC1 and PC2.

Data analysis was performed in R, using the packages *multilevel* for Cronbach’s alpha scores, *factoextra* for principal component analysis, and *ggplot2* and *sjPlot* for visualization (Bliese, 2016; Wickham, 2016; Lüdecke, 2018; Kassambara and Mundt, 2020; R Core Team, 2020).

## Results

The average donation was AUD $6.10 (SD: $3.56). Without controlling for covariates, the average donation for the high-impact message was $6.83 (SD: $3.91), compared to $5.83 (SD: $3.40) for the medium-impact message and $5.59 (SD: $3.36) for the low-impact message.

The outcome of this experimental study provides preliminary evidence as to how climate organizations can capture greater funding by drawing on findings from scientific research to craft an inspiring message. A boost of 25%, from the low- to the high-impact message, as calculated from model predictions, is substantial when considered across the fundraising efforts of a climate organization or the entire sector (Bergquist et al., 2020). The funding that climate and environmental organizations currently receive is far short of what is necessary to achieve solutions (Osuri, 2010; Waldron et al., 2013; Bergquist et al., 2020; Ryba and Connell, 2020). Achieving a boost in private donations for a minimal investment of effort can be part of the answer.

## Discussion

Evidence can be a powerful motivator for change. There is much progress remaining for society to reach the level of funding necessary to achieve meaningful climate solutions (Yeo, 2019; Nature Climate Change, 2020). Fundraisers and the climate organizations that they support have the opportunity to adopt an evidence-based viewpoint on how to captivate donors, drawing upon the evolving literature on philanthropic studies (Bekkers and Wiepking, 2010; Whillans, 2016) and climate psychology (Weber, 2010, 2016; Center for Research on Environmental Decisions [CRED] and EcoAmerica, 2014). The evidence in our experimental study shows that the financial returns for doing so could be substantial. This provides optimism that the non-profit sector can inspire donors to contribute much-needed resources toward climate solutions.

There are a number of avenues by which our experimental study can be improved. Firstly, our sample size was quite low, at 70 observations across three treatment groups. While this number satisfied our initial power analysis, we were expecting far more participants. However, the university at which participants were recruited was closed due to the COVID-19 pandemic in between experimental sessions. Indeed, many members of the research community have had to reconsider study design and adjust expectations for this reason (Barroga and Matanguihan, 2020; Coleman et al., 2020). The small sample size increases the risk of overinterpreting the data, particularly given the interaction effects and inclusion of three PCs. We included the interaction effects based on previous theoretical and empirical studies on this topic. Likewise, we retained three PCs based on the criterion of Lott (1973). However, this criterion (as with every decision rule for retaining PCs) is imperfect. When the interaction terms or PC3 are dropped, for example, the effect sizes remain similar, but the statistical significance does not. For this reason, we strongly encourage the interpretation of this study as a preliminary foundation for future work with larger sample sizes.

Secondly, and relatedly, a small number of our participants were given an online version of the experiment with rewards that were hypothetical, rather than real. Adapting studies to online platforms is another change that many researchers have had to make (Garcia and Barclay, 2020; Hussain, 2020; Vicente et al., 2020). The statistical results are very similar when this subgroup is excluded (see Supplementary Information). Thirdly, manipulation checks may ensure that participants understood the information presented to them. This would help ensure that the treatments are meaningful and effective in bringing about the intended changes (Hauser et al., 2018). Fourthly, the summary statistics of participant demographics reveal that the sample was typical for a university campus, but not necessarily representative of potential audiences of climate organizations (see Supplementary Information). Given these limitations, we encourage the interpretation of our experimental study as a preliminary step, and we anticipate future studies that take further steps down these avenues toward improving the methodology.

Here, we showed how adopting a broad toolkit from published literature may boost donations to a climate organization. To help organizations make the most of this opportunity, researchers can unpack the science of effective climate appeals at a finer scale. Published studies in that context, using real money, are few (Milinski et al., 2006; Blasch, 2014; Ellis et al., 2016; Löschel et al., 2017, 2013; Diederich and Goeschl, 2018; Goeschl et al., 2018; Svenningsen, 2019). Openings remain for providing insight into precisely what inspires donors to contribute to climate solutions. We believe that this is an important avenue for future research—providing detailed insight for climate organizations to engage with donors can help capture greater donations, which in turn can help address the resource gap in the societal challenge that is addressing climate change.

In our experimental study, we provided donors with the opportunity to provide open-ended feedback. This revealed one further avenue by which climate charities can be aided: building trust. Several participants expressed skepticism of charities, with comments such as “Charities are rife with misuse of funds,’ and ‘I often find myself skeptical of a charity’s merits.” This skepticism mirrors issues with trust and accountability in the not-for-profit sector as a whole (Bourassa and Stang, 2016; Kantar Public, 2017). The emergence of *effective altruism*, a movement that promotes donating to causes and organizations supported by rigorous, scientific evidence, provides one way for charities to demonstrate their effectiveness (MacAskill, 2015; Singer, 2015). Indeed, scientific evidence of a charity’s effectiveness has been shown to increase donations (Vollan et al., 2017), and we selected the charity in this study for its performance in a systematic assessment of climate charities (Halstead, 2018). Educating donors about the scientific evidence for a charitable organization may be a critical step toward restoring donors’ trust and generosity.

## Data Availability Statement

The raw data supporting the conclusions of this article will be made available by the authors, without undue reservation.

## Ethics Statement

The studies involving human participants were reviewed and approved by School of Psychology Human Research Ethics Committee at the University of Adelaide. The participants provided their written informed consent to participate in this study.

## Author Contributions

RR and SC performed investigation. RR performed data curation, formal analysis, and wrote original draft. All authors contributed to review, editing, conceptualization, and methodology.

## Conflict of Interest

The authors declare that the research was conducted in the absence of any commercial or financial relationships that could be construed as a potential conflict of interest.

## Publisher’s Note

All claims expressed in this article are solely those of the authors and do not necessarily represent those of their affiliated organizations, or those of the publisher, the editors and the reviewers. Any product that may be evaluated in this article, or claim that may be made by its manufacturer, is not guaranteed or endorsed by the publisher.

## Footnotes

1 We selected this organization as it ranked as a highly effective, evidence-based climate charity at the time of the study. More recent evidence has revised the conclusion as to this charity’s cost-effectiveness. We encourage interested readers to seek the latest recommendations for cost-effective charities, available from many organizations including Founders Pledge (www.founderspledge.com).
