# Supplementary material for: Climate Donations Inspired by Evidence-Based Fundraising
Source: Front Psychol. 2022 Mar 7;13:768823. doi: 10.3389/fpsyg.2022.768823 (PMC8936950; doi:10.3389/fpsyg.2022.768823)
Supplement: Supplementary file 1 [file Data_Sheet_1.docx]

Supplementary Material

# Tables and Figures

Table 1: We constructed three messages asking for a donation to a climate charity, at three levels of impact. * denotes information removed for anonymity purposes.

| **High-impact** | A major problem facing the world today is climate change. This problem has been linked to higher rates of disease for people living in Australia by 2020. In fact, over two-thirds of university students are seriously concerned about this issue. Furthermore, the risk is becoming more urgent with each year.  We are asking you to make a donation to The Coalition for Rainforest Nations. This charity’s mission is to mitigate climate change by promoting forestry programs. This charity has been recommended by Dr **** ********, a leading policy researcher from Oxford University.  Each dollar you donate directly averts 8 tonnes of carbon dioxide through advocacy for forestry programs. Your money will also boost employment in developing countries. By donating, you can earn a feeling of satisfaction.  Kind regards,  The Coalition for Rainforest Nations |
| --- | --- |
| **Medium-impact** | A major problem facing the world today is climate change. This problem has been linked to higher rates of disease for people living in Latin America by 2100. In fact, some members of the community are seriously concerned about this issue. Furthermore, the risk is becoming more urgent with each year.  We are asking you to make a donation to The Coalition for Rainforest Nations. This charity’s mission is to mitigate climate change by promoting forestry programs. This charity has been recommended by Dr **** ********, a leading policy researcher from Oxford University.  Each dollar you donate contributes to averting emissions through advocacy for forestry programs. Your money will operate in developing countries. By donating, you can earn a feeling of satisfaction.  Kind regards,  The Coalition for Rainforest Nations |
| **Low-impact** | A major problem facing the world today is air pollution and its impact on human health. This problem has been linked to higher rates of disease for people living in Latin America by 2100. In fact, some members of the community are seriously concerned about this issue. Furthermore, research is conducted on this topic each year.  We are asking you to make a donation to The Coalition for Rainforest Nations. This charity’s mission is to reduce air pollution by promoting forestry programs. This charity was the subject of a recent blog post.  Each dollar you donate contributes to averting emissions through advocacy for forestry programs. Your money will operate in developing countries. By donating, you can help preserve our environment.  Kind regards,  The Coalition for Rainforest Nations |

Table 2: To collect data on participants’ demographics, beliefs and worldviews, we used these survey questions and scales. All scales are expressed on a scale from 1 to 5. The Australian political parties include Liberal-National Coalition (centre-right), Labor (centre-left), Greens (environmental), and One Nation (far right).

| Donor characteristics | Reference | Mean (SD) | Cronbach’s alpha |
| --- | --- | --- | --- |
| Age |  | 22.4 (5.23) | – |
| Gender |  | Female: 60.0% of participants  Male: 38.6% of participants  Non-binary: 1.43% of participants | – |
| Political interest | (McAllister et al., 2017) | 3.37 (1.25) | – |
| Stated position on political spectrum (1 = left; 5 = right) | (McAllister et al., 2017) | 2.19 (0.982) | – |
| Stated support for major political parties | (McAllister et al., 2017; Milfont et al., 2012) | Liberal-National Coalition: 2.07 (0.890)  Labor: 3.40 (0.841)  Greens: 4.00 (0.901)  One Nation: 1.50 (0.776) | – |
| Calculated support for major political parties’ policies | (Vox Pop Labs, n.d.) | Liberal-National Coalition: 2.47 (0.381)  Labor: 3.12 (0.423)  Greens: 3.07 (0.795)  One Nation: 1.70 (0.387) | – |
| Postmaterialist index | (Inglehart and Abramson, 1999) | Materialist: 1.43% of participants  Mixed: 64.3% of participants  Postmaterialist: 34.3% of participants | – |
| Financial well-being | (Ranta and Salmela-Aro, 2018) | 2.98 (0.838) | 0.718 |
| Subjective financial situation | (Ranta and Salmela-Aro, 2018) | 3.37 (1.04) | – |
| Individual-communitarian cultural worldview (1 = communitarian; 5 = individualist) | (Kahan et al., 2012; Shi et al., 2015) | 3.06 (0.488) | 0.610 |
| Hierarchy-egalitarian cultural worldview (1 = egalitarian; 5 = hierarchical) | (Kahan et al., 2012; Shi et al., 2015) | 1.91 (0.717) | 0.834 |
| Climate change concern | (Shi et al., 2015) | 4.67 (0.416) | 0.802 |
| Religious beliefs and values | (King et al., 2006) | 2.48 (0.761) | 0.941 |
| Self-reported altruism scale | (Philippe Rushton et al., 1981) | 2.79 (0.524) | 0.842 |

Table 3: The statistical model when the hypothetical subgroup was excluded. **p* < 0.05.

|  | **donation** | | | | |
| --- | --- | --- | --- | --- | --- |
| *Predictors* | *Estimates* | *std. Error* | *t* | *p* |  |
| Intercept | 12.42 | 5.57 | 2.23 | **0.030** |  |
| Impact | -4.70 | 2.62 | -1.80 | 0.079 |  |
| PC1 | -0.14 | 5.57 | -0.03 | 0.980 |  |
| PC2 | -17.76 | 8.95 | -1.99 | 0.053 |  |
| PC3 | -4.70 | 7.86 | -0.60 | 0.553 |  |
| PC1*Message | 1.58 | 3.00 | 0.52 | 0.602 |  |
| PC2*Message | 9.20 | 4.05 | 2.27 | **0.028** |  |
| PC3*Message | 3.44 | 3.37 | 1.02 | 0.312 |  |
| Observations  df | 55  47 | | | | |
| R^2^ / R^2^ adjusted | 0.162 / 0.037 | | | | |
| AIC | 305.382 | | | | |


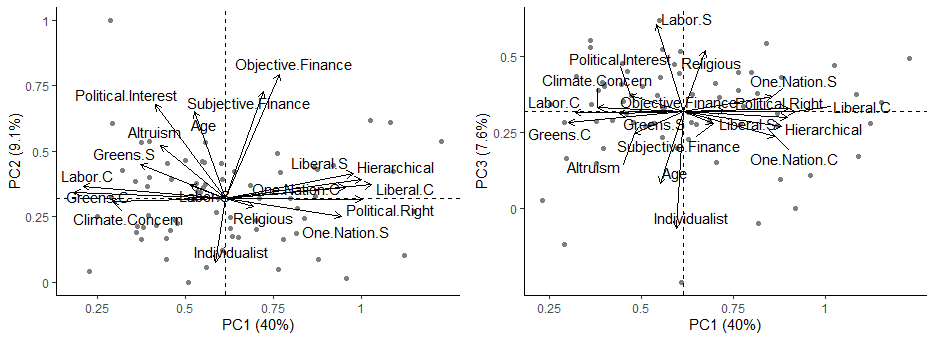


Figure 1: We corrected for donors’ personal characteristics by performing principal component analysis on donor characteristics. We retained three components (PC1, PC2, PC3). Points show participants’ positions, and lines show the beliefs and characteristics that compose the components. (C = calculated support for a political party’s policies; S = stated support for that party.)

# References

Inglehart, R., Abramson, P.R., 1999. Measuring Postmaterialism. American Political Science Review 93, 665–677. https://doi.org/10.2307/2585581

Kahan, D.M., Peters, E., Wittlin, M., Slovic, P., Ouellette, L.L., Braman, D., Mandel, G., 2012. The polarizing impact of science literacy and numeracy on perceived climate change risks. Nature Clim Change 2, 732–735. https://doi.org/10.1038/nclimate1547

King, M., Jones, L., Barnes, K., Low, J., Walker, C., Wilkinson, S., Mason, C., Sutherland, J., Tookman, A., 2006. Measuring spiritual belief: development and standardization of a Beliefs and Values Scale. Psychol. Med. 36, 417–425. https://doi.org/10.1017/S003329170500629X

McAllister, I., Makkai, T., Bean, C., Gibson, R., 2017. Australian Election Study, 2016 [WWW Document]. URL http://dx.doi.org/10.4225/87/7OZCZA

Milfont, T.L., Harré, N., Sibley, C.G., Duckitt, J., 2012. The Climate-Change Dilemma: Examining the Association Between Parental Status and Political Party Support1. Journal of Applied Social Psychology 42, 2386–2410. https://doi.org/10.1111/j.1559-1816.2012.00946.x

Philippe Rushton, J., Chrisjohn, R.D., Cynthia Fekken, G., 1981. The altruistic personality and the self-report altruism scale. Personality and Individual Differences 2, 293–302. https://doi.org/10.1016/0191-8869(81)90084-2

Ranta, M., Salmela-Aro, K., 2018. Subjective financial situation and financial capability of young adults in Finland. International Journal of Behavioral Development 42, 525–534. https://doi.org/10.1177/0165025417745382

Shi, J., Visschers, V.H.M., Siegrist, M., 2015. Public Perception of Climate Change: The Importance of Knowledge and Cultural Worldviews. Risk Analysis 35, 2183–2201. https://doi.org/10.1111/risa.12406

Vox Pop Labs, n.d. Vote compass methodology.
